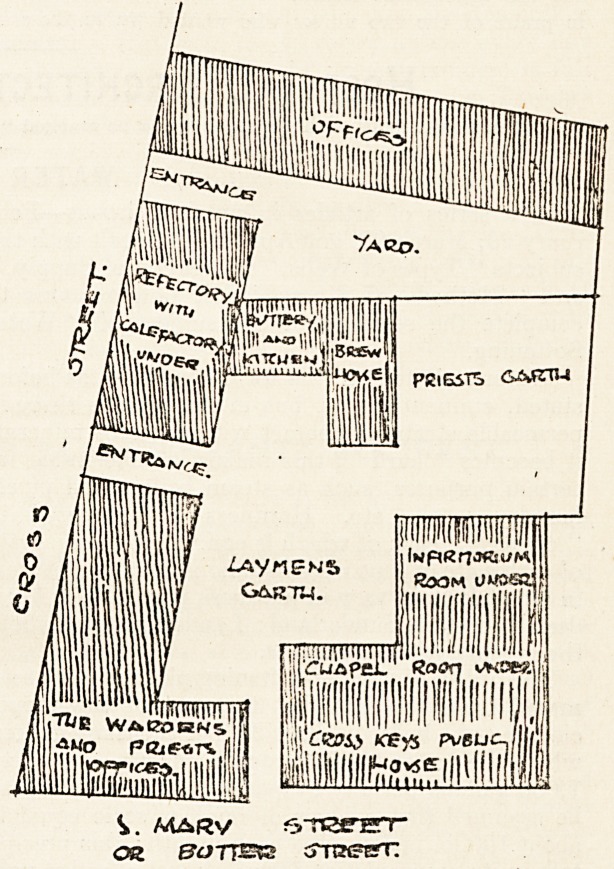# A Mediæval Hospital

**Published:** 1911-06-17

**Authors:** 


### A MEDIÆVAL HOSPITAL.

The accompanying sketches are of a building originally erected by Bishop Henry de Gower in 1330 in Swansea. The front faced St. Mary Street, running east and west, and contained the Hospital Chapel. The Charter relates that "The said Master or Warden of the said hospital and the chaplains for the time being and the other poor persons dwelling therein as aforesaid do celebrate (services) for the soul of our late Lord David, Bishop of St. David's, etc."

The main buildings comprised in the plan of the Hospital of the Blessed David appear to have been an irregular quadrangle enclosing two garths, a brewhouse and kitchen with domestic offices. Of the two garths one was probably used as a kitchen or herb garden for the laity; the other was set apart for the priests. The southern side of the present building appears to have been occupied by the chapel and infirmarium, with offices under. At the south-west corner would most likely be the wardens' and priests' lodgings, and in the building beyond, on the west side, the refectory; it evidently having been open to the roof, a portion of which still exists as in the other portion of the buildings. Under this probably would be the calefactory or general meeting-room for talk, etc. To the northern side would be the kitchen, the brewhouse adjoining it.

The foregoing gives an outline of this very important semi-monastic establishment. There would appear to have been an entrance from the west side to the large garth, and near this an aperture in the wall which might be the buttery or serving-window for giving out doles.

We are indebted to Glendinning Moxham, F.R.I.B.A., of Swansea, for our archæological information.

## Figures and Tables

**Figure f1:**
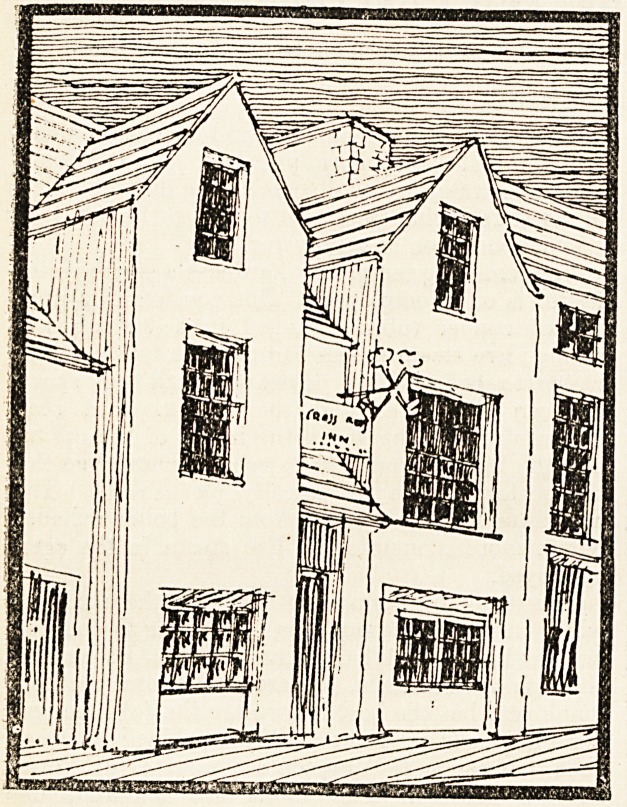


**Figure f2:**